# Diverse Sublethal Effects of a Common Fungicide Impact the Behavior and Physiology of Honey Bees

**DOI:** 10.3390/insects16060603

**Published:** 2025-06-08

**Authors:** Xufeng Zhang, Qian Cao, Feng Wang, Yinyin Du, Wen Zhao, Yuan Guo, Olav Rueppell

**Affiliations:** 1 College of Horticulture, Shanxi Agricultural University, Taiyuan 030001, China; zhangxf@sxau.edu.cn; 2 College of Animal Sciences, Shanxi Agricultural University, Jinzhong 030801, China; caoqian_0423@163.com (Q.C.); fengwang0224@163.com (F.W.); duyinyin032200@163.com (Y.D.); 3 Institute of Apicultural Research, Chinese Academy of Agricultural Sciences, Beijing 100193, China; zhaowen@caas.cn; 4 Department of Biological Sciences, University of Alberta, Edmonton, AB T6G 2L3, Canada

**Keywords:** carbendazim, compound fungicide, gut microbiome, memory, metabolome, propiconazole, risk assessment, sublethal effects

## Abstract

Honey bees and other pollinators are key to functioning natural and managed ecosystems. However, their health is threatened by many factors, including pesticides. Spraying fungicides during flowering of fruit trees is widespread even though it directly exposes pollinators to these fungicides. Here, we report a series of experiments designed to understand how the combination of propiconazole and carbendazim, marketed in China as Chunmanchun^®^, affects honey bee health. With an acute oral toxicity of 23.8 μg a.i./bee over 24 h in the laboratory, we considered the acute mortality risk from normal Chunmanchun^®^ applications as relatively low. However, our comprehensive studies revealed other diverse effects: Chunmanchun^®^ reduced memory after classic conditioning by approximately 25% and altered the activity of protective enzymes and the composition of the honey bees’ gut microbiota. Specifically, the genus *Lactobacillus* was decreased by ~13%, and *Bartonella* and *Snodgrassella* were increased by ~10% and ~7.5%, respectively. The gut metabolome was also disrupted in diverse ways, possibly as a functional consequence of the microbiome changes. Thus, we demonstrated numerous sublethal effects of the combination of propiconazole and carbendazim, which adds to the growing evidence that agrochemicals and fungicides in particular can harm pollinator health in subtle ways that are not captured in simple mortality assays.

## Simple Summary

Pollinators like honey bees are vital for healthy ecosystems and successful crop production, but their health is under threat from various factors, including overused agricultural chemicals. This study focused on understanding how a commonly used fungicide mixture called Chunmanchun^®^, sprayed during the flowering of pear trees in China, affects honey bees. While the chemical did not cause immediate death when bees consumed it in small amounts, we found several sublethal effects on bee health. Bees exposed to Chunmanchun^®^ had a 25% reduction in memory, which could affect their ability to find food and return to the hive. The chemical also disrupted important protective enzymes and changed the balance of bacteria in their digestive systems, reducing helpful bacteria and increasing potentially harmful ones. These changes also affected the bees’ metabolism, which may further impact their health. This research highlights that even when pesticides do not cause immediate death, they can still harm bees in subtle but important ways. These findings indicate the need for a more careful evaluation of how agricultural chemicals affect pollinators, especially compounds that are used when pollinators are most active. Only with more comprehensive testing can bee populations be protected to maintain healthy ecosystems and food supplies.

## 1. Introduction

Pollinators, including wild and managed bees, play a crucial role in pollinating many natural and agricultural plants [1,2,3]. Approximately 20,000 identified species of bees are key pollinators [4], and 35% of worldwide food production relies on their pollination services [5]. Bee pollination ensures food security and is also important for the functioning of many terrestrial ecosystems [6]. However, populations of bees and other pollinators are declining worldwide, which has prompted concerted efforts to understand the potential causes and consequences of pollinator decline and better promote pollinator health [7,8].

Current agriculture relies on the extensive use of pesticides to lessen the immediate impacts of pests or weeds on crop production [9]. However, the extensive use of pesticides exposes beneficial species, such as bees and other pollinators, to damaging chemicals and may therefore have unintended side effects [9,10]. Pollination requires mutualistic interactions between crops and bees, thus exposing the insect pollinators directly to a range of fungicides, herbicides, and insecticides [11]. The use of pesticides in agricultural landscapes has been identified as a significant stressor that can negatively affect bee health [12,13,14].

Numerous studies have demonstrated the detrimental effects of agrochemicals on bees, including reduced foraging activity [15], impaired cognitive function [16,17,18], and increased susceptibility to diseases [19,20,21]. Exposure of honey bees to sublethal doses of pesticides can disrupt navigation [22], communication [23], and learning abilities [24], ultimately affecting their ability to pollinate crops effectively [25,26]. However, comprehensive studies are rare and every agrochemical can have different effects and even different formulations; moreover, application methods may vary.

Research interest in fungicide impacts on honey bees has consequently increased in recent years [13,26,27,28]. Compound formulations can invalidate the safety evaluation of individual compounds because they can have additional negative effects. For example, Pristine^®^, a mixture of pyraclostrobin and boscalid, can cause protein deficiency and malnutrition and disturb the gut microbiota of bees [29]. Probably as a consequence, Pristine^®^ also results in earlier transitioning to foraging and shortened lifespan of worker honey bees [30]. Furthermore, it can impair the olfactory associative learning performance of honey bees [31]. Alone, pyraclostrobin has developmental and immunological effects [32,33], while boscalid has been shown to decrease the flight performance of adult workers [34] but had minor effects on larval and adult gene expression and survival [35]. Thus, the effects of compound fungicides need to be separately evaluated from their constituents.

A compound fungicide consisting of a 7% propiconazole and 28% carbendazim suspension–emulsion (Chunmanchun^®^, Zhaoyuan Sanlian Chemical Co., Ltd., Zhaoyuan, Shandong province, China), which is itself an emulsifiable concentrate, is in widespread use during the pear blossom period in China’s Shanxi Province to control pear disease and achieve increased calyx removal in young pear fruits. Spraying during active honey bee foraging exposes foraging workers directly to the fungicide mixture and may also contaminate collected food resources. Propiconazole is a broad-spectrum systemic triazole fungicide with protective and therapeutic effects against bacteria and fungi [36]. Propiconazole is effective against a range of pests and its high activity, fast but long-lasting action, and systemic effects make it highly effective [37]. Carbendazim is also an efficient, broad-spectrum systemic fungicide with protective and therapeutic effects [38]. However, the impact of the compound formulation of propiconazole and carbendazim on different aspects of the health of honey bees is unclear and we studied Chunmanchun^®^ with the objective to comprehensively assess its effects on bee health.

In a series of field and laboratory experiments, this study assessed the effects of spraying Chunmanchun^®^ during the pear blossom period on the foraging behavior and physiology of worker honey bees as a comprehensive evaluation of its risks to honey bee health. First, we characterized the dynamics of bee mortality and fungicide residues across a field application of propiconazole/carbendazim. Second, we evaluated acute toxicity and found a low risk for honey bees based on the Chinese national criteria for acute toxicity testing. However, we found that memory, protective enzyme activity, and the gut microbiome and metabolome of exposed honey bees were affected. This study will provide a basis for the study of the ecotoxicological effects of compound fungicides on bees and provide an important data reference for ensuring the safety of bees and guiding pear farmers in the rational and safe use of fungicides.

## 2. Materials and Methods

All experiments were conducted with honey bees (*Apis mellifera ligustica*) raised at the Institute of Horticulture, Shanxi Agricultural University, Taiyuan, Shanxi Province, China (37° 48′ 6” N, 112° 36′ 16” E, altitude 795 m) in March to September of 2023. Each colony was free of signs of disease and contained an egg-laying queen and seven full-sized combs of workers, which had sufficient honey and pollen.

We performed acute contact and oral toxicity tests to inform a risk assessment of Chunmanchun^®^. To study potential sublethal effects, we used the proboscis extension reflex to assess the impacts of Chunmanchun^®^ exposure on learning and memory. Additionally, we evaluated the activity of protective and detoxifying enzymes between the exposed and control bees. Finally, we characterized the gut microbiome communities and compared the metabolomes of bees that were exposed to either Chunmanchun^®^ or a control treatment. The details of all the chemical reagents used in our experiments were shown as below the Table 1. 

### 2.1. Reagents

### 2.2. Acute Toxicity Tests

#### 2.2.1. Acute Contact and Oral Toxicity Testing

After collection of foragers that were returning to the hive, the bees were housed in a dark, climate-controlled room (25 ± 2 °C, 50–70% relative humidity) in wooden boxes with a Plexiglas top (10 cm × 30 cm × 5 cm) and fed with 50% (wt:wt) sucrose water until satiation. After 30 min, dead and other non-responsive individuals were removed and all remaining bees were starved for 2 h.

Contact toxicity was measured after a topical application of 116 μg of Chunmanchun^®^ per bee in ultrapure water. A negative control was included and bees were exposed to a serial dilution of 98% dimethoate (0.360, 0.277, 0.213, 0.164, and 0.126 g/L) as the positive control by dripping 1 μL onto the scutum of the mesonotum of individual bees.

To test oral toxicity, the positive control of 98% dimethoate was dissolved in acetone and then diluted with 50% sugar solution to concentrations of 11.78 × 10^−3^, 9.60 × 10^−3^, 7.41 × 10^−3^, 5.23 × 10^−3^, and 3.49 × 10^−3^ g/L. In parallel, Chunmanchun^®^ was diluted in a 50% sucrose solution to 3.50, 1.75, 0.875, 0.583, and 0.438 g/L. Along with a 50% sucrose solution as the negative control, the effect of orally feeding 150 μL of these solutions was tested. All solutions were consumed in about 4 h, although we cannot exclude the possibility that some solution was only stored in the bees’ crops.

Each treatment and the control group had three replicates (independent hives), with 10 bees in each replicate. The survival was monitored for 48 h under dark conditions in wooden boxes with a Plexiglas top (10 cm × 30 cm × 5 cm) in an incubator with a temperature of 25 ± 2 °C and a relative humidity of 50.0–70.0% while feeding the bees with 50% sucrose solution ad libitum. The number of bee deaths in each group was determined 24 h and 48 h after exposure. Death was defined as the complete cessation of all movement and one of the following symptoms: wings spread, abdomen flexed, and/or proboscis extended. In the acute oral toxicity and acute contact toxicity tests, each concentration was tested in three replicates with 10 bees per replicate.

#### 2.2.2. Toxicity Evaluation

Based on the number of bees that died after 24 h and 48 h for the tests of different concentrations, a probit regression was used to calculate the semi-lethal dose (LD_50_) and its 95% confidence limits from the regression equation.

#### 2.2.3. Risk Assessment

The hazard quotient (HQ) for contact and oral toxicity was calculated as the ratio of the recommended dosage of pesticides in the field (g/hm^2^) to the respective LD_50_ values (μg a.i./bee) [39,40,41,42].

### 2.3. The Proboscis Extension Reflex Experiment

To test the effects of the fungicide mixture on the cognitive function of worker bees, individuals were trained to associate citral with a sucrose reward by olfactory conditioning of the proboscis extension reflex (PER) after Chunmanchun^®^ exposure or control treatment. Foragers were captured as described above and randomly divided into an experimental and a control group and maintained in the above-described boxes for treatment. Chunmanchun^®^ was diluted to 0.159 g/L (the recommended concentration for field application) in 50% sucrose solution and 6 mL of this solution was fed to groups of 30 bees. This could have maximally allowed for an average consumption of 31.8 µg Chunmanchun^®^ per bee over 24 h although actual consumption was likely much lower. An equal number of control bees were fed with 50% sucrose solution only during the same time period before a 30 min starvation period that preceded the training for all bees. The PER set-up and training routine followed previously described protocols with each bee individually harnessed to restrict body movements [43]. Twenty bees were used in each group and the experiment was performed three times with bees from independent replicate hives.

Bees were anesthetized with CO_2_ and fixed inside a transparent plastic straw (diameter: 6 mm) and housed in an incubator (35 °C and 65% relative humidity), the fixed bees were starved for 0.5 h before the experiment. Bees that responded spontaneously to air puffs delivered from a mounted, clean syringe or behaved abnormally in other ways were excluded. All remaining bees were then fed with 50% sucrose solution for 3 s and bees with positive PER to 50% sucrose were selected. Another syringe containing 2 µL citral [44] filter paper was mounted above the antennae of the fixed bee to allow the delivery of puffs of air at a constant speed for 4 s. With 1 s overlap with the delivery of citral as the conditioned stimulus, the antennae of the bee were touched with 50% sucrose solution (unconditioned stimulus) for 3 s. A proboscis extension in response to citral in the first 3 s was recorded as a positive response. If the response only occurred after the delivery of the 50% sucrose solution, the trial was recorded as a negative response to citral. Each round of trials was separated by 5 min during which the space was completely ventilated. The harnessed bees were conditioned for six trials of paired citral odor–sucrose presentations with an average inter-trial interval of 10 min. Memory tests were performed after 1 h, 3 h, and 6 h after the training period by presenting citral without a sucrose reward, and bees that responded with an extension of their proboscis were scored as memory positive. During this time, bees remained in their harnesses and were not fed. No deaths occurred. Learning and memory at the different time points were compared between fungicide-exposed and control bees with 2 × 2 contingency tests.

### 2.4. Enzyme Activity Testing

To investigate the potential sublethal effects of the fungicide at the biochemical level, the activity of several important detoxification enzymes was measured after exposure of newly emerged bees to the compound fungicide. A first experiment used exposure of the field-recommended dose over 3–72 h, while a second experiment compared the impact of 24 h exposure at three different concentrations and a negative control. The three concentrations were selected based on the recommended concentration for field application, and the LD_10_ and LD_50_ as determined above. The LD_10_ and LD_50_ are above the recommended field application dose, but we included these dosages nevertheless because individual bee exposure to such concentrations in the field cannot be excluded. Each experiment was performed with three replicates each with 30 bees from a separate colony.

For both experiments, the capped comb was placed in a dark incubator with a temperature of 32 (±2) °C and relative humidity of 50~70%. After 12 h, newly emerged bees were collected from their comb and starved for an additional 2 h in the incubator before initiating the experiments.

In the first experiment, the Chunmanchun^®^ suspoemulsion was diluted in 50% (m/m) sucrose solution to 0.159 g/L, and 50% sucrose solution was used as the control. Three replicate cages per experimental group for each time point with thirty bees each were housed together in an incubator and after mass-feeding for 3, 6, 12, 24, 48, and 72 h (6 mL sucrose solution every 24 h), all surviving bees were collected, anesthetized with CO_2_, immediately snap-frozen in liquid nitrogen, and stored in a −80 °C freezer for catalase (CAT), superoxide dismutase (SOD), peroxidase (POD), carboxylesterase (CarE), glutathione S-transferases (GSTs) and cytochrome P450 (CYP450) enzyme solution preparation. In the second experiment, Chunmanchun^®^ was diluted to 0.159 g/L, 0.250 g/L, 0.583 g/L, and with 50% sucrose solution, and bees were fed as described above. Surviving bees were collected after 24 h, anesthetized with CO_2_, immediately frozen in liquid nitrogen, and stored in a −80 °C freezer. In all instances, the sucrose solution was completely consumed, resulting in average dosing of individuals that directly correspond to the different treatments.

Solarbio^®^ Enzyme Activity Assay Kits (Beijing Solarbio Sciences & Technology Co., Ltd., Beijing, China) were used for testing the enzymatic activity of CAT, SOD, POD, CarE, GSTs, and CYP450. From each bee, 0.1 g of abdominal tissue was added to 1 mL of extraction solution and homogenized thoroughly on ice, then centrifuged at 8000× *g* and 4 °C for 10 min. The supernatant was collected and placed on ice for CAT, SOD, POD, CarE, and GST activity detection. For CYP450, 0.1 g of abdominal tissue was added to 0.5 mL PBS, pH 7.4, homogenized thoroughly on ice, and centrifuged at 3000 rpm and 4 °C for 20 min, after which the supernatant was collected and placed on ice. Enzymatic activities were quantified by exactly following the manufacturer’s instructions accompanying the enzyme kit; for CYP450, the absorbance value at 450 nm was measured. For SOD, after the enzyme solution to be tested was fully mixed with the reagents in the kit, followed by a 37 °C water bath for 30 min, it was placed in a 1 mL glass cuvette to measure the absorbance at 560 nm. For POD, it was placed in a 1 mL glass cuvette to measure the absorbance at 470 nm for 30 s and 90 s. For CAT, 1 mL of CAT detection working solution was placed in a 1 mL glass cuvette, and then 35 μL of the enzyme solution to be tested was added, mixed for 5 s, and the initial absorbance was immediately measured at 240 nm and the absorbance was measured after 1 min at room temperature. For CarE, the absorbance values at 450 nm were measured for 10 s and 310 s. For GSTs, the absorbance change was measured at 340 nm, and the absorbance values at 10 s and 310 s were recorded. Each treatment was performed three times. After an overall evaluation of the effects of treatment and time (using a General Linear Model), we performed simple effect tests where necessary to assess treatment effects at each time point via t tests with Bonferroni correction.

### 2.5. Analysis of Gut Microbiota

#### 2.5.1. Sample Preparation

Newly emerged worker bees were marked with non-toxic paint (Tamiya, Ikegami Paint Industry Company, Neuss Germany) and returned to their original colonies. After seven days, the marked bees were recaptured and randomly divided into four experimental groups that were exposed to the same concentrations as used for enzyme activity testing: the control group (CK), the recommended field concentration (PC) group, and the estimated LD_10_ and LD_50_ groups, with 30 bees in each group and each of three hive replicates (total *N* = 360).

The CK group was fed with 50% sucrose water (m/m) solution, while the PC, LD_10_, and LD_50_ groups were fed with Chunmanchun^®^ diluted with 50% sucrose water solution to 0.159 g/L, 0.308 g/L, and 1.011 g/L, respectively. Each bee received 0.2 mL of sucrose solution per day for 6 days.

After exposure, worker bees were quickly frozen, surface-sterilized with 75% alcohol, and then rinsed with a sterile PBS solution. The complete digestive tract was pulled out of each bee with pointed tweezers, the venom sacs were removed, and a glass rod was used to push out all the gut content. From each group, 15 bees were selected and combined into three biological replicates (5 intestines per biological replicate). The pooled intestines were placed in an Eppendorf™ tube (Eppendorf AG, Hamburg, Germany), immediately frozen in liquid nitrogen, and stored in sealed bags at −80 °C.

#### 2.5.2. DNA Extraction and PCR Amplification

Total microbial genomic DNA was extracted from samples using the E.Z.N.A.^®^ soil DNA Kit (Omega Bio-tek, Norcross, GA, USA) according to the manufacturer’s instructions. The quality and concentration of DNA were determined by 1.0% agarose gel electrophoresis and a NanoDrop^®^ ND-2000 spectrophotometer (Thermo Scientific Inc., Waltham, MA, USA) and stored at −80 °C. The hypervariable region V3-V4 of the bacterial 16S rRNA gene was amplified with primers 338F (5′-ACTCCTACGGGAGGCAGCAG-3′) and 806R (5′-GGACTACHVGGGTWTCTAAT-3′) [45] using an ABI GeneAmp^®^ 9700 PCR thermocycler (ABI, Los Angeles, CA, USA). The PCR reaction mixture included 4 μL 5 × Fast Pfu buffer, 2 μg/L, 2.5 mM dNTPs, 0.8 μL of each primer (5 μM), 0.4 μL Fast Pfu polymerase, 10 ng of template DNA, and ddH_2_O added to make the final volume of 20 µL. PCR amplification cycling conditions were as follows: initial denaturation at 95 °C for 3 min, followed by 27 cycles of denaturation at 95 °C for 30 s, annealing at 55 °C for 30 s, and extension at 72 °C for 45 s, followed by a single extension step at 72 °C for 10 min and indefinite storage at 4 °C. All samples were amplified in triplicate. The PCR products were extracted from a 2% agarose gel and purified using the AxyPrep DNA Gel Extraction Kit (Axygen Biosciences, Union City, CA, USA) according to the manufacturer’s instructions and quantified using Quantus™ Fluorometer (Promega, Madison, WI, USA).

#### 2.5.3. Illumina MiSeq Sequencing

Purified amplicons were pooled in equimolar amounts and paired-end sequenced on an Illumina MiSeq PE300 platform (Illumina, San Diego, CA, USA) according to the standard protocols by Majorbio Bio-Pharm Technology Co., Ltd. (Shanghai, China). The raw sequencing reads were deposited into the NCBI Sequence Read Archive (SRA) database (Accession Number: SRP499770).

#### 2.5.4. Amplicon Sequence Processing and Analysis

After demultiplexing, the resulting sequences were quality-filtered with fastp (version 0.19.6) [46] and merged with FLASH (v1.2.11) [47]. The sequences were then de-noised using the DADA2 [48] plugin in the Qiime2 (version 2020.2) pipeline [49] with default parameters, resulting in single nucleotide resolution sequences. DADA2 denoised sequences are here referred to as amplicon sequence variants (ASVs) and represent our primary level of analysis. To minimize the effects of sequencing depth on alpha and beta diversity estimates, the number of sequences from each sample was rarefied to 20,000, which yielded an average Good’s coverage of 97.90%. Taxonomic assignment of ASVs was performed using the Naive Bayes consensus taxonomy classifier implemented in Qiime2 and the SILVA 16S rRNA database (v138).

Bioinformatic analysis of the gut bacteria communities of bees was carried out using the Majorbio Cloud platform (https://cloud.majorbio.com, accessed on 1 July 2023). Based on the observed ASVs, alpha and beta diversity indices were calculated [50]. The sample differences were visualized by principal coordinate analysis (PCoA) based on the Bray–Curtis dissimilarity metric using the Vegan v2.5-3 package. A PERMANOVA test was used to assess the overall effects of treatment using the Vegan v2.5-3 package. A linear discriminant analysis (LDA) effect size [51] was determined to identify the significantly abundant taxa (phylum to genera) of bacteria among the different groups (LDA score > 2, *p* < 0.05).

### 2.6. Effects of Different Concentrations of Compound Fungicide on Intestinal Metabolites in the Hindgut of Bees

Potential effects of the Chunmanchun^®^ exposure on the metabolic profile of the bees’ hindgut were assessed by untargeted metabolomic profile comparisons between exposed and control bees. Newly emerged bees were collected, marked, and returned for 7 days to their hive as described above. Upon recollection, they were treated with the same three concentrations of Chunmanchun^®^ that were used to assess gut microbiome disruptions (PC, LD_10_, and LD_50_) or left untreated as the control (*n* = 9 pools of 5 bees each per experimental group). The gut was extracted as described above (Section 2.5.1). From each bee, 50 mg was added to a 2 mL centrifuge tube, and then a 6 mm diameter grinding bead was added. An amount of 400 μL of extraction solution (methanol: water = 4:1 (*v*:*v*)) containing 0.02 mg/mL of internal standard (L-2-chlorophenylalanine) was used for metabolite extraction. The samples were ground by a Wonbio-96c (Shanghai Wanbo Biotechnology Co., LTD) frozen tissue grinder for 6 min (−10 °C, 50 Hz), followed by low-temperature ultrasonic extraction for 30 min (5 °C, 40 kHz). The samples were left at −20 °C for 30 min, centrifuged for 15 min (4 °C, 13,000 g), and the supernatant was transferred to the injection vial for LC-MS/MS analysis.

As a part of the system conditioning and quality control process, a pooled quality control sample (QC) was prepared by mixing equal volumes of all samples. The QC samples were treated and tested in the same manner as the analytic samples. The QC sample was injected at regular intervals (every 5–15 samples) to monitor the stability of the analysis.

The LC-MS/MS analysis was conducted on a Thermo UHPLC-Q Exactive HF-X system equipped with an ACQUITY HSS T3 column (100 mm × 2.1 mm i.d., 1.8 μm; Waters, USA) at Majorbio Bio-Pharm Technology Co., Ltd. (Shanghai, China). The mobile phases consisted of 0.1% formic acid in water: acetonitrile (95:5, *v*/*v*) (solvent A) and 0.1% formic acid in acetonitrile: isopropanol: water (47.5:47.5:5, *v*/*v*/*v*) (solvent B). A positive ion mode separation gradient was performed with the following profile: 0–3 min with mobile phase A and increasing mobile phase B from 0% to 20%; 3–4.5 min—mobile phase B increasing from 20% to 35%; 4.5–5 min—mobile phase B increasing from 35% to 100%; 5–6.3 min—mobile phase B maintained at 100%; 6.3–6.4 min—mobile phase B decreasing from 100% to 0%; and between 6.4 and 8 min the mobile phase B was maintained at 0%. The same sample was then analyzed by a separation gradient in negative ion mode: mobile phase B was rising from 0 to 5% (0–1.5 min,), 5% to 10% (1.5–2 min), 10% to 30% (2–4.5 min), and 30% to 100% (4.5–5 min); maintained at 100% (5–6.3 min); decreasing from 100% to 0% (6.3–6.4 min); and maintained at 0% (6.4–8 min). The flow rate was maintained at 0.40 mL/min, and the column temperature was 40 °C.

The MS conditions were as follows: The mass spectrometric data were collected using a Thermo UHPLC-Q Exactive HF-X Mass Spectrometer (Thermo Scientific Inc., Waltham, MA, USA) equipped with an electrospray ionization (ESI) source operating in positive mode and negative mode. The optimal conditions were set as follows: source temperature at 425 °C; sheath gas flow rate at 50 arb; Aux gas flow rate at 13 arb; ion-spray voltage floating (ISVF) at −3500 V in negative mode and 3500 V in positive mode, respectively; and normalized collision energy, 20–40–60 V rolling for MS/MS. Full MS resolution was 60,000, and MS/MS resolution was 7500. Data acquisition was performed with the Data Dependent Acquisition (DDA) mode. The detection was carried out over a mass range of 70–1050 M/Z.

The pretreatment of LC/MS raw data was performed in the Progenesis QI software (Waters Corporation, Milford, CT, USA). The metabolites were identified by searching the databases HMDB (http://www.hmdb.ca/, accessed on 1 July 2023), Metlin (https://metlin.scripps.edu/, accessed on 1 July 2023), and Majorbio (https://www.majorbio.com/, accessed on 1 July 2023).

Using the Majorbio cloud platform (https://cloud.majorbio.com, accessed on 1 July 2023), the data matrix was preprocessed, retaining only variables (peaks corresponding to individual metabolites) with less than 20% non-zero values. Zero values were subsequently replaced with the value of the lower detection limit. Variables with a relative standard deviation of >30% among quality control samples were considered unreliable and deleted. For each sample, the sum of all peaks was calculated and used to normalize each peak’s value. The remaining data were log10-transformed for subsequent analysis. The R package “ropls” (Version 1.6.2) was used to perform principal component analysis (PCA) and partial least squares discriminant analysis (PLS-DA), and 7-cycle interactive validation was performed to evaluate the stability of the model. The metabolites with variable importance in the projection (VIP > 1, *p* < 0.05) were determined as significantly different metabolites based on the VIP obtained by the orthogonal partial least squares discriminant analysis (OPLS-DA) model with *p*-values generated by Student’s *t* tests.

Differential metabolites among the two groups were mapped into their biochemical pathways through metabolic enrichment and pathway analysis based on the KEGG database (http://www.genome.jp/kegg/, accessed on 1 July 2023). Python packages “scipy.stats” V1.15.3 (https://docs.scipy.org/doc/scipy/, accessed on 1 July 2023) were used to perform enrichment analyses to obtain the most relevant biological pathways affected by the experimental treatments.

### 2.7. Data Analyses

All data were collected using Microsoft Excel™ and analyzed using SPSS™ 27.0 and 29.0 (IBM, Armonk, NY, USA). Unless specified otherwise, we performed Pearson’s correlations and standard ANOVAs with Tukey tests for post hoc analyses when data did not significantly deviate from parametric assumptions. For count data, Kruskal–Wallis tests were performed as a non-parametric alternative without post hoc testing, but PER performance was evaluated with repeated measures ANOVA. Multiple testing correction for KEGG pathways enrichment was performed with the Benjamini–Hochberg procedure.

## 3. Results

### 3.1. Toxicity Determination and Risk Assessment of Chunmanchun^®^

**Figure 1:**
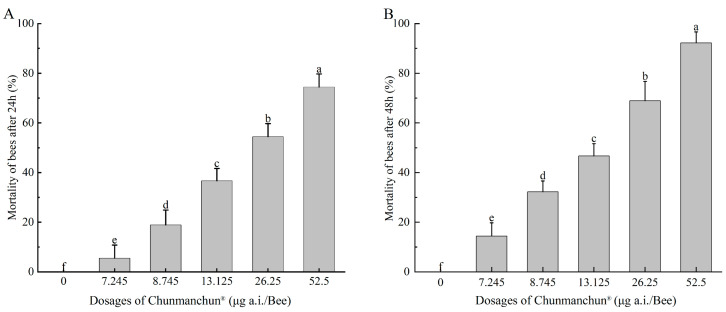
Mortality of honey bee workers exposed to different dosages of oral Chunmanchun^®^ exposure. (**A**) Mortality of bees treated with different dosages of the fungicide after 24 h. (**B**) Mortality rates of bees in response to different dosages of the fungicide after 48 h. Different lowercase letters indicate significant differences at the 0.05 level as determined by ANOVA with Tukey tests for post hoc analyses. Mean ± SD values are plotted.

In the acute contact toxicity test, no obvious phenotypic abnormalities were found within 48 h after bees had been exposed to Chunmanchun^®^. The LD_50_ over 48 h was estimated to exceed 116 μg active ingredient per bee and therefore no hazard quotient could be calculated. The acute oral LD_50_ of Chunmanchun^®^ was estimated as 23.8 μg a.i./bee (95% confidence limit: 20.7–28.1 μg a.i./bee) over 24 h and 15.2 μg a.i./bee (95% CI: 13.5–17.2 μg a.i./bee) over 48 h (Figure 1, Supplementary Table S1). Conservatively, the lowest LD_50_ was used together with the field recommended dose of 749.63 g/hm^2^ to calculate a hazard quotient of 49.42, indicating a low risk for worker honey bees.

### 3.2. Effects of Chunmanchun^®^ on Learning and Memory of Bees

**Figure 2:**
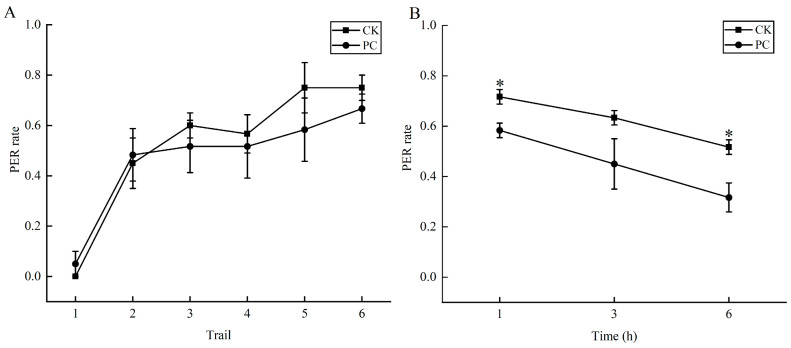
Effects of Chunmanchun^®^ on learning and memory of honey bee workers. (**A**) The proportion of honey bees exhibiting a proboscis extension reflex (PER) during six successive classic conditioning trials was not significantly different between the Chunmanchun^®^-treated (PC) and control (CK) groups (repeated measures ANOVA, *n* = 120, with 3 independent replicates of 20 bees per group each). (**B**) Recall tests showed a consistently lower performance of the PC than the CK group, but the difference was only significant after one and six hours (* represents *p* < 0.05).

The learning performance of bees exposed to Chunmanchun^®^ was not significantly different from the control group at any point in the acquisition phase (*F*_(5,114)_ = 1.2, *p* = 0.390). The recall ability (memory) of the same individuals was not significantly different (*F*_(5,114)_ = 17.3, *p* = 0.053) between the control and treatment group after three hours (Figure 2A; Supplementary Table S2), but the control bees outperformed the Chunmanchun^®^-treated bees after one hour (*F*_(5,114)_ = 49.0, *p* = 0.020) and six hours (*F*_(5,114)_ = 30.3, *p* = 0.032; Figure 2B; Supplementary Table S3).

### 3.3. Effects of Chunmanchun^®^ on Protective and Detoxifying Enzymes

**Figure 3:**
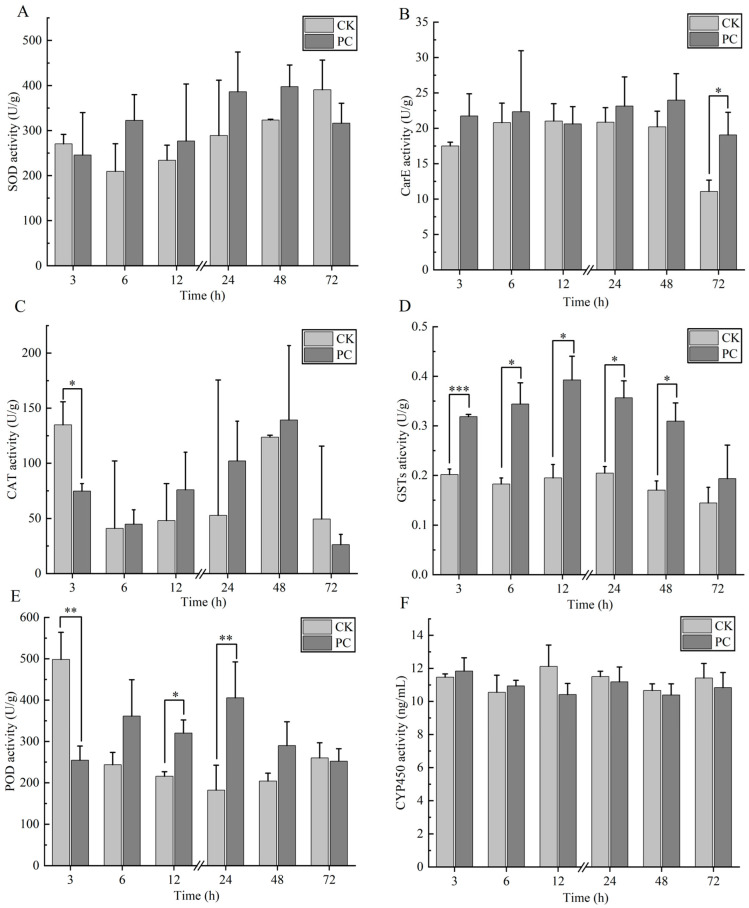
Effects of Chunmanchun^®^ on select enzyme activities across time. (**A**) Superoxide dismutase (SOD), (**B**) carboxylesterase (CarE), (**C**) catalase (CAT), (**D**) glutathione S-transferases (GSTs), (**E**) peroxidase (POD), and (**F**) cytochrome P450 (CYP450). Mean ± SD values from 3 replicates are displayed. At each time point, an independent-sample t test was used for each enzyme. “*” represents *p* < 0.05; “**” represents *p* < 0.01; and “***” represents *p* < 0.001. CK = control and PC = exposure to Chunmanchun^®^ at the field-recommended concentration of 0.159 g/L.

The effects of Chunmanchun^®^ on enzyme activities were complex and varied among enzymes (Figure 3; Supplementary Table S4). Superoxide dismutase (SOD) activity (Figure 3A) was not affected by treatment (*F*_(1,24)_ = 2.4, *p* = 0.135) but varied among time points (*F*_(5,24)_ = 2.8, *p* = 0.040) with no interaction between the two factors (*F*_(5,24)_ = 1.5, *p* = 0.229). Carboxylesterase (CarE) activity (Figure 3B) was affected by treatment (*F*_(1,24)_ = 7.2, *p* = 0.013) and time (*F*_(5,24)_ = 3.3, *p* = 0.022) but not their interaction (*F*_(5,24)_ = 0.9, *p* = 0.476) and none of the pairwise comparisons between the treatment and control groups at specific time points were significant after Bonferroni correction. Catalase (CAT) activity (Figure 3C) was significantly different among time points (*F*_(5,24)_ = 7.9, *p* < 0.001), but no significant treatment (*F*_(1,24)_ = 0.04, *p* = 0.837) or interaction effects (*F*_(5,24)_ = 2.3, *p* = 0.080) were present. The activity of glutathione S-transferases (GSTs) was significantly affected by treatment (*F*_(1,24)_ = 48.1, *p* < 0.001) and time (*F*_(5,24)_ = 3.4, *p* = 0.018), but not their interaction (*F*_(5,24)_ = 1.1, *p* = 0.385). GST activity was significantly higher in treated than control samples after 3 h (*t* = 9.8, *P_adj_* = 0.004), while the differences at all other time points were consistent with this result but not significant after Bonferroni correction (Figure 3D). The peroxidase activity (POD) was significantly affected by treatment (*F*_(1,24)_ = 7.2, *p* = 0.013) and time (*F*_(5,24)_ = 4.9, *p* = 0.003), as well as their interaction (*F*_(5,24)_ = 14.2, *p* < 0.001). POD activity was significantly lowered by treatment after 3 h (*t* = −5.7, *P_adj_* = 0.03) but increased after 24 h (*t* = 5.4, *P_adj_* = 0.036; Figure 3E), while differences at all other time points were not significant after Bonferroni correction. No significant differences of cytochrome P450 (CYP450) activity were found with respect to treatment (*F*_(1,24)_ = 1.9, *p* = 0.182), time (*F*_(5,24)_ = 1.7, *p* = 0.173), or interaction (*F*_(5,24)_ = 1.5, *p* = 0.234; Figure 3F).

**Figure 4:**
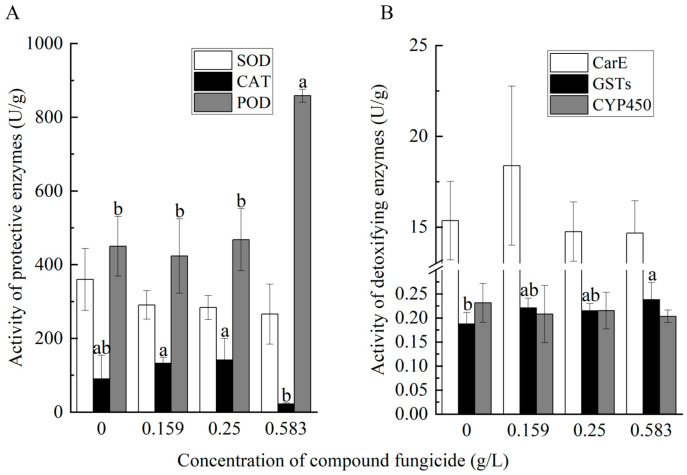
Effects of Chunmanchun^®^ on selected enzyme activities over different concentrations. (**A**) The activities of superoxide dismutase (SOD), catalase (CAT), and peroxidase (POD) of bees were tested with different concentrations of compound fungicide. (**B**) The activities of carboxylesterase (CarE), glutathione S-transferases (GSTs), and cytochrome P450 (CYP450) of bees were tested with different concentrations of compound fungicide. Each enzyme was compared with a separate ANOVA, followed by LSD tests for pairwise comparisons. Mean ± SD values are depicted based on *n* = 3. Different lowercase letters indicate significant differences at the 0.05 level.

When the effect of different concentrations on these enzyme activities was measured after 24 h exposure, SOD (*F*_(3,8)_ = 1.3, *p* = 0.109), CarE, and CYP450 did not show any significant effects (Figure 4), while POD activity was significantly increased (*F*_(3,8)_ = 21.1, *p* < 0.001) and CAT activity was decreased (*F*_(3,8)_ = 4.6, *p* = 0.011) by the highest Chunmanchun^®^ concentration (Figure 4A) and GSTs increased gradually from the lowest to the highest concentration (*F*_(3,8)_ = 2.1, *p* = 0.038; Figure 4B; Supplementary Table S5).

### 3.4. Gut Microbiome Effects of Compound Fungicide Exposure

A total of 2,451,894 high-quality sequences with a total of 1.04 Gbps were generated from our 36 samples (9 replicates of the control and each Chunmanchun^®^ concentration). Among the sequences, 1248 bacterial amplicon sequence variants (ASVs) were identified at the 97% sequence similarity cut-off (Supplementary Table S6). Good’s coverage index indicated that our average bacterial coverage across samples was 0.99 ± 0.00026 (mean ± SD). Visual inspection of the rarefaction curves confirmed that the sequencing effort adequately captured the gut microbiome communities.

**Figure 5:**
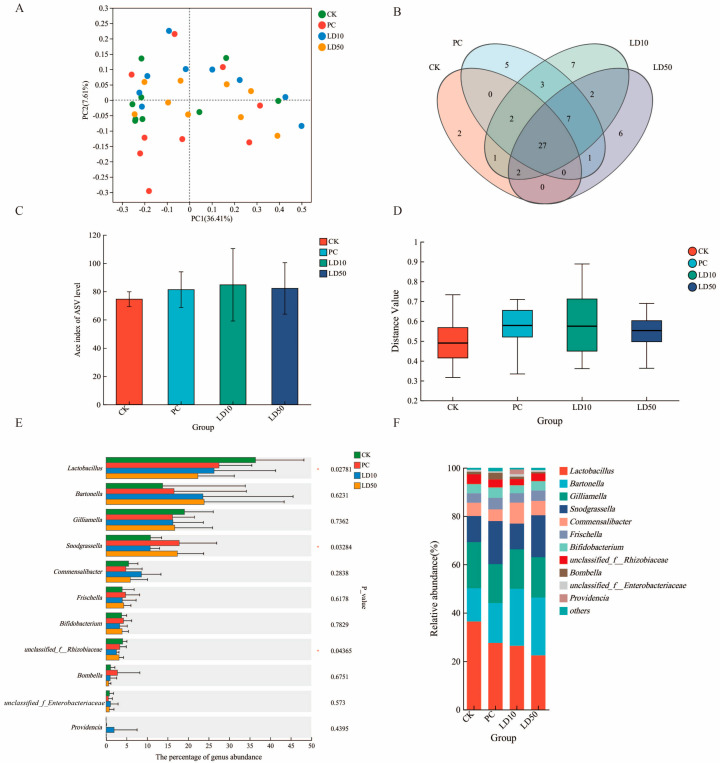
Analysis of the gut microbiome of honey bee workers after treatment with different concentrations of the compound fungicide Chunmanchun^®^. The four concentrations correspond to the negative control (CK), the field recommended dose (PC: 0.159 g/L), the LD_10_ (0.308 g/L), and the LD_50_ (1.01 g/L) groups. Each of these groups contained nine replicates. (**A**) Principal coordinates analysis showed little separation of the four different treatment groups along the first and second principal coordinates. (**B**) Venn diagram analysis at the genus classification level indicated most taxa overlapping, but also some unique genera in treatment groups. (**C**) Alpha diversity of the gut microbiome, computed as the ace index, was not significantly different among treatment groups (*p* = 0.816). (**D**) Beta diversity of the gut microbiome was significantly different among treatment groups (*p* = 0.025). (**E**) The abundances of four of eleven genera of the gut microbiome were significantly different (* represents *p* < 0.05) among groups. (**F**) Summary of relative abundances of genera in (**E**), illustrating the consistent dose-dependent response of *Bartonella* (positive) and *Lactobacillus* (negative).

Across all four treatment groups, we identified 1248 ASVs in 83 species, representing 60 genera, 39 families, and 27 orders in ten distinct classes. A principal coordinate analysis demonstrated only a small degree of overall differentiation between the treatment groups along the first and second principal coordinates, which accounted for 36.41% and 7.41% of the total variance, respectively (Figure 5A). At the genus level, 27 taxa were shared among all treatment groups as opposed to 20 taxa that occurred only in one of the groups (two for the control, five for the recommended field dose, seven for LD_10_, and six for LD_50_ (Figure 5B). The alpha diversity of the bee gut microbiota was not significantly different among treatments (Kruskal–Wallis: H = 0.94, *n* = 36, *p* = 0.816; Figure 5C; Supplementary Table S7). In contrast, beta diversity was significantly different among treatment groups (*p* = 0.025; Figure 5D).

Differences among treatments were significant for three of the eight most abundant genera (*Lactobacillus*, *p* = 0.028; *Snodgrassella*, *p* = 0.033; and Rhizobiaceae unclassified genus, *p* = 0.044), and strict dose-dependency was observed in *Lactobacillus* and *Bartonella* (negative and positive, respectively; Figure 5E and Figure 5F). In contrast, no significant differences were exhibited by the other genera (*Bartonella*, *p* = 0.623; *Gilliamella*, *p* = 0.736; *Commensalibacter*, *p* = 0.284; *Frischella*, *p* = 0.618; and *Bifidobacterium*, *p* = 0.783) (Supplementary Table S8). *Providencia* sp. was only identified in samples treated with the LD_10_ concentration of Chunmanchun^®^.

### 3.5. Hindgut Metabolome Effects of Compound Fungicide Exposure

**Figure 6:**
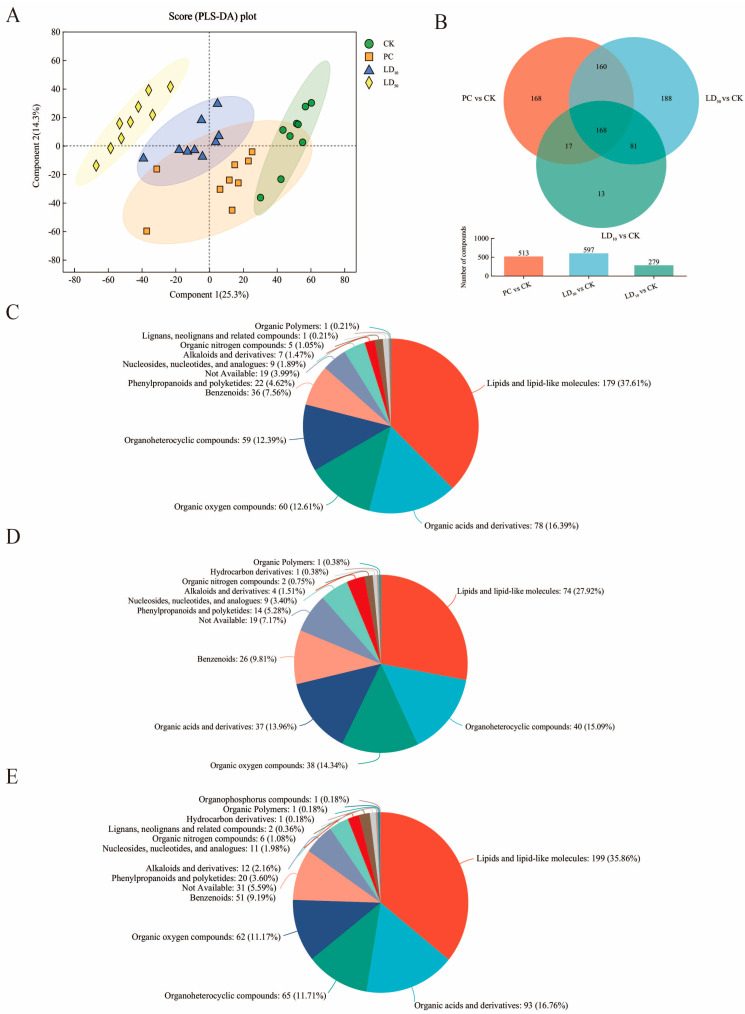
Metabolomic profiling of the hindgut of honey bee workers treated with different concentrations of Chunmanchun^®^. (**A**) The partial least squares discriminant analysis plot based on the complete metabolomics dataset displays a separation of all four treatments with different concentrations (PC = 0.159 g/L, LD_10_ = 0.308 g/L, LD_50_ = 1.011 g/L, indicated by different colors according to the legend, QC = quality control, each *n* = 9). (**B**) Venn diagram of different comparison groups of metabolites of the hindgut of bees treated with different concentrations of compound fungicide. (**C**) Compound classification of differential metabolites between the PC and CK groups. (**D**) Compound classification of differential metabolites between the LD_10_ and CK groups. (**E**) Compound classification of differential metabolites between the LD_50_ and CK groups.

To understand the physiological consequences of Chunmanchun^®^, an untargeted metabolomic analysis of hindgut samples from workers exposed to CK, PC, LD_10_, and LD_50_ treatments was performed. A total of 3857 raw metabolites were identified after data preprocessing, with 3421 metabolites in all four treatments, including organic acids and derivatives, lipids, amino acids, nucleic acids, benzenoids, organoheterocyclic compounds, organonitrogen compounds, organic oxygen compounds, organosulfur compounds, phenylpropanoids, and polyketides (Supplementary Table S9). A partial least squares discriminant analysis revealed a separation among the four treatment groups, along with principal components 1 and 2, which accounted for 24.7% and 22.7% of the total variance, respectively (Figure 6A).

Metabolites with VIP values > 1.0 in the partial least squares discriminant analysis and a Student’s t test *p* < 0.05 were considered statistically significantly different in pairwise comparisons among experimental groups. Overlap analysis indicated that 168 differential metabolites were shared among different comparisons, while the PC vs. CK and LD_50_ vs. CK comparisons also revealed large numbers of unique differences in metabolites (Figure 6B).

The 513 differential metabolites from the comparison of PC and CK (Supplementary Table S10) included 179 lipids (37.61%), 78 organic acids and derivatives (16.39%), 60 organic oxygen compounds (12.61%), 59 organoheterocyclic compounds (12.39%), and 36 benzenoids (7.56%; Figure 6C). Between LD10 and CK, 279 differential metabolites were identified (Supplementary Table S11), including 74 lipids (27.92%), 40 organoheterocyclic compounds (15.09%), 38 organic oxygen compounds (14.34%), 37 organic acid and derivatives (13.96%), and 26 benzenoids (9.81%; Figure 6D). The comparison of LD_50_ and CK identified 597 differential metabolites (Supplementary Table S14), including 199 lipids (35.86%), 93 organic acids and derivatives (16.76%), 65 organoheterocyclic compounds (11.71%), 62 organic oxygen compounds (11.17%), and 51 benzenoids (9.19%; Figure 6E). In all comparisons, Chunmanchun^®^ treatment led to a quantitatively more pronounced up-regulation of metabolites than down-regulation, while there was no clear numerical difference in how many metabolites were up- or down-regulated (Supplementary Tables S10–S12).

**Figure 7:**
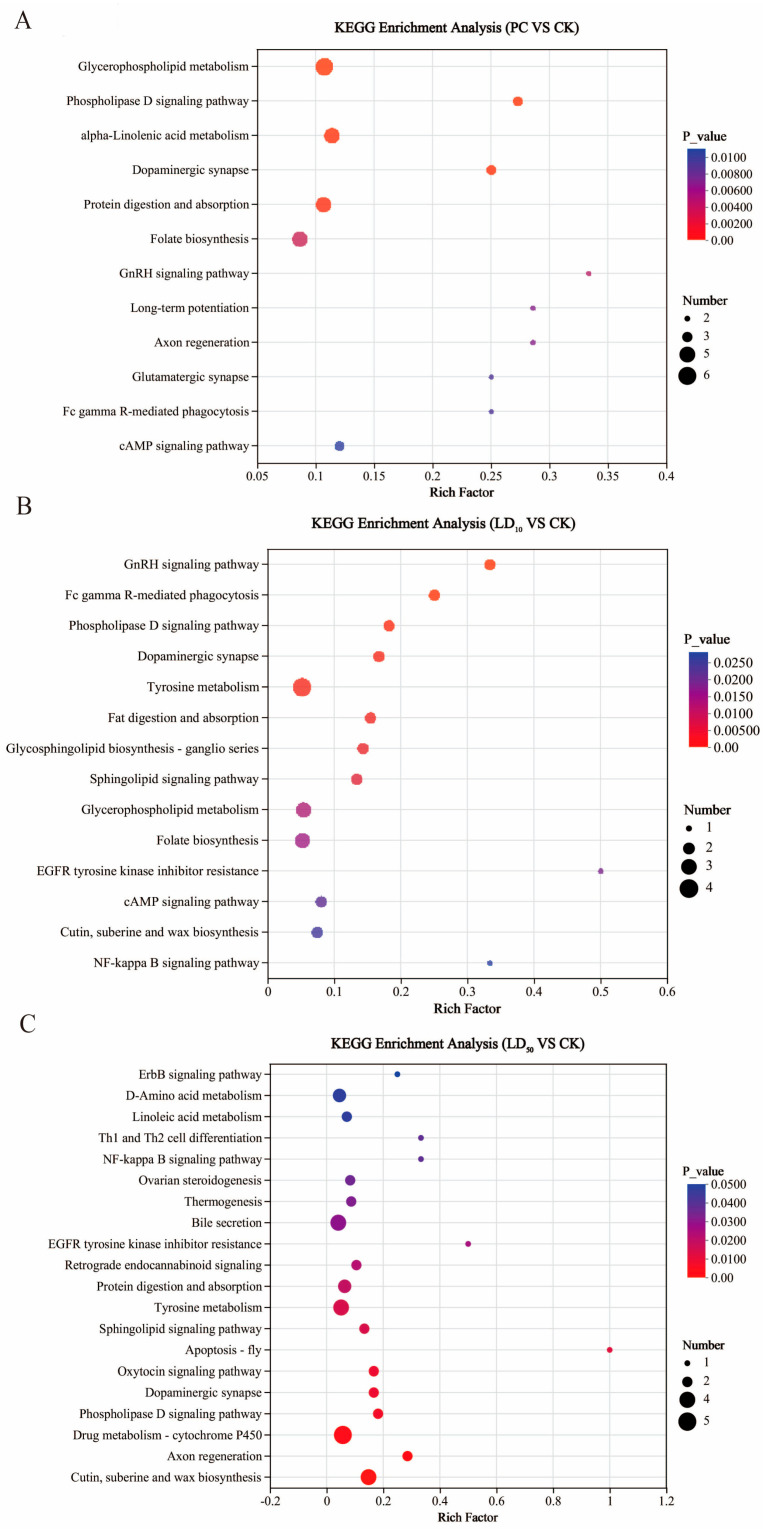
KEGG pathway enrichment of differential metabolites of the hindgut of honey bee workers treated with different concentrations of Chunmanchun^®^ compared to the control group. (**A**) The KEGG pathway enrichment of differential metabolites in the PC and CK groups. Multiple testing correction: Benjamini–Hochberg. (**B**) The KEGG pathway enrichment of differential metabolites in the LD_10_ and CK groups. Multiple testing correction: Benjamini–Hochberg. (**C**) The KEGG pathway enrichment of differential metabolites in the LD_50_ and CK groups. Multiple testing correction was performed according to the Benjamini–Hochberg procedure.

To further characterize the functional changes in the gut metabolome in response to Chunmanchun^®^, KEGG pathway enrichment analysis of differentially abundant metabolites was performed. In the comparison of PC to CK, 36 KEGG pathways were significantly enriched among the 513 differential named metabolites (*p* < 0.05). The enriched pathways were primarily involved in lipid metabolism (“Glycerophospholipid metabolism”), signal transduction (“cAMP signaling pathway”), neuronal function (“Dopaminergic synapse”, “Glutamatergic synapse”, “Axon regeneration”), immunity (“Fc gamma R-mediated phagocytosis”), digestive system (“Protein and fat digestion and absorption”), amino acid metabolism (“Glycine, serine and threonine metabolism”), and the endocrine system (“GnRH signaling pathway”; Figure 7A). All enriched pathway functions (Supplementary Table S13) were significantly increased by the PC treatment, except “Tyrosine metabolism”.

Comparing the LD_10_ and CK treatments, we found that 279 differential metabolites were significantly enriched in 36 KEGG pathways (*p* < 0.05; Supplementary Table S14), which were primarily involved in lipid metabolism (“Glycerophospholipid metabolism”, “Cutin, suberine and wax biosynthesis”), signal transduction (“cAMP signaling pathway”), metabolism of cofactors and vitamins (“Folate biosynthesis”), glycan biosynthesis and metabolism (”Glycosphingolipid biosynthesis”), amino acid metabolism (“Tyrosine metabolism”), nervous system (“Dopaminergic synapse”), and digestive system (“Fat digestion and absorption”). Among these enriched pathways, only the “Cutin, suberine and wax biosynthesis pathway” was significantly down-regulated (*p* = 0.025), the remaining significantly enriched pathways were up-regulated (Figure 7B).

For the comparison of the LD_50_ exposure group to the control, CK, 597 differential named metabolites were significantly (*p* < 0.05) enriched in 38 KEGG pathways (Supplementary Table S15), including lipid metabolism (“Glycerophospholipid metabolism”), amino acid metabolism (“Glycine, serine and threonine metabolism”, “Tyrosine metabolism”), metabolism of cofactors and vitamins (“Folate biosynthesis”), immune system (“Fc gamma R-mediated phagocytosis”), xenobiotics biodegradation and metabolism (“Drug metabolism-CYP450”), nervous system (“Glutamatergic synapse”, “Dopaminergic synapse”, “Serotonergic synapse”), digestive system (“Protein digestion and absorption”), cell growth and death (“Apoptosis”), translation (“Aminoacyl-tRNA biosynthesis”), and environmental adaptation (“Circadian entrainment”). Among these, pathways related to signal transduction, xenobiotics biodegradation and metabolism, “Cutin, suberine and wax biosynthesis” “Tyrosine metabolism”, “Apoptosis”, “EGFR tyrosine kinase inhibitor resistance” and “Axon regeneration” were significantly down-regulated, while all others were up-regulated (Figure 7C).

### 3.6. Relation Between Gut Microbiome and Metabolites

**Figure 8:**
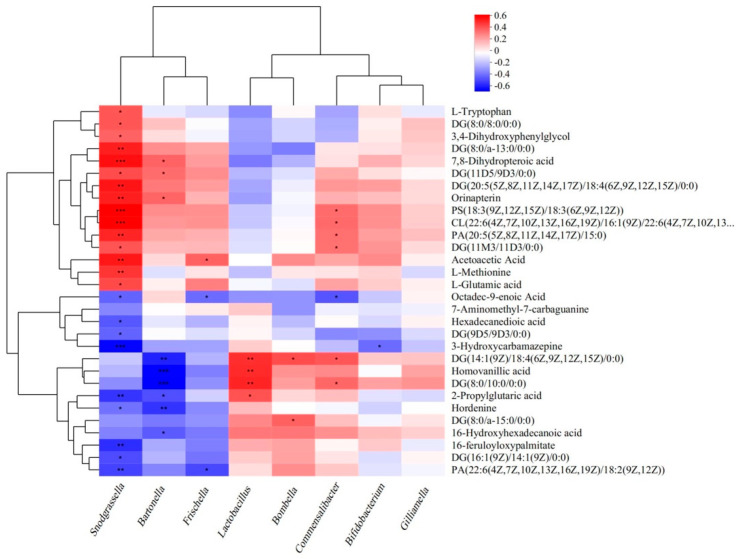
Correlation analysis between key metabolites and bacterial core genera in the honey bee gut. Most of the significant correlations with 30 metabolites belonging to the enriched KEGG pathways were found for *Snodgrassella*, indicating a central role of this genus in the hindgut metabolism of honey bees. However, all genera except *Gilliamella* were significantly correlated with some of the metabolites and *Lactobacillus* exhibited a complementary pattern to *Snodgrassella*. Color intensities indicate the magnitude of the correlation coefficient (Pearson’s), ranging from negative (blue) to positive (red). Spearman correlations. “*” represents *p* < 0.05, “**” represents *p* < 0.01, and “***” represents *p* < 0.001.

In search for binary relations between the bees’ gut microbiome and metabolome, we correlated the relative abundance of eight core bacteria and 30 metabolites that were related to the most enriched KEGG pathways and were negatively affected by Chunmanchun^®^ in a dose-dependent manner (Figure 8). The results indicated that none of these metabolites were significantly correlated to *Lactobacillus*, *Bombella*, *Bifidobacterium*, or *Gilliamella*.

*Snodgrassella* abundance was positively correlated to metabolites mostly involved in “Folate biosynthesis”, “Tyrosine metabolism”, “Glycerophospholipid metabolism”, pathways related to signal transduction, protein and fat digestion and absorption pathways, signal transduction, and lipid metabolism. *Snodgrassella* abundance was negatively correlated to metabolites involved in “Fat digestion and absorption”, “cAMP signaling pathway”, “Fc gamma R-mediated phagocytosis pathways”, and “drug metabolism-CYP450”. *Bartonella* also showed significant correlations to several metabolites, most strikingly negative relations with signaling pathways, “Drug metabolism-CYP450”, and “Tyrosine metabolism”. *Frischella* and *Commensalibacter* exhibited only a few weak relations.

## 4. Discussion

Our study of different biological effects of the compound fungicide Chunmanchun^®^ suggests that this agrochemical can have unintended effects on honey bees, similar to many other studies that have documented negative impacts of agrochemicals on beneficial organisms [25,52]. Despite the low acute toxicity of Chunmanchun^®^ in the laboratory, we identified memory deficiency, changes in enzyme activities, gut microbiome disruption, and widespread impacts on the metabolome of the hindgut of worker honey bees. However, the social colony environment can buffer individuals from stress effects [53] and we did not measure any colony-level effects of Chunmanchun^®^ treatment. Therefore, we were unable to determine whether Chunmanchun^®^ has any measurable detrimental effects on colony growth and performance under field conditions.

The acute contact and oral toxicity of Chunmanchun^®^ measured in our laboratory experiments was low. According to both LD_50_ values and application guidelines, it can be classified as “low toxicity”, the lowest toxicity classification based on the “Chemical Pesticide Environmental Safety Assessment Test Guidelines Part 10: Acute Toxicity Test on Bees” (GB/T 31270.10-2014), which provides national standards in China. LD_50_ values are widely used to calculate hazard quotients as indicators of the ecological risk of pesticides to bees. Conservatively, our lowest LD_50_ of 15.2 μg a.i./bee for oral Chunmanchun^®^ exposure can be translated into a hazard quotient of 49.4 by combination with the field recommended dose of 749.6 g/hm^2^, which would indicate a low risk for worker honey bees. However, this does not provide conclusive evidence that Chunmanchun^®^ is harmless because discrepancies between laboratory and field trials are not uncommon [30,54]. Such discrepancies may be explained by relatively few deaths among the many thousands of worker bees that are exposed, which cannot be adequately reflected in laboratory assays. The removal of food resources from the hive before colony placement, a common practice to enhance pollination in the studied system, could increase mortality as well and pesticide exposure under natural conditions can result in more severe outcomes due to the interaction with other stressors [55,56]. Lastly, this result could also suggest that exposure in the field is heterogeneous, resulting in mortality of particularly exposed or vulnerable individuals [57,58]. Regardless of the mechanistic explanation, any discrepancy between laboratory and field mortality studies in conjunction with the sublethal effects that are further discussed below raises the question of whether standard toxicological evaluation of agrochemicals in the laboratory is sufficient to assess their risks to non-target organisms in a complex ecological environment. In honey bees, this concern is amplified because different castes of the colony may differ in susceptibility.

Protective enzymes play an important role in maintaining physiological homeostasis after toxin exposure, shielding cells and macromolecules from potential harm [59]. Despite the bee’s smaller repertoire of these enzymes, their activity responds to pesticide exposure in specific ways [60,61,62], which was confirmed for Chunmanchun^®^ here. For CAT and POD, an initial inhibition 3 h after exposure was followed by a recovery that turned into a significantly increased activity after 12 h for POD. In contrast, GST function was significantly increased by Chunmanchun^®^ treatment across all time points except 72 h. At that time point, there was a significant increase in CarE activity in treated relative to control bees, but the functional relevance of this difference is unclear. Presumably due to small sample sizes, the experiment did not reveal any other significant treatment effects and we could not confirm that sublethal doses of carbendazim induce SOD activity in bees [63]. The CAT and POD effects indicate that propiconazole and/or carbendazim induce oxidative stress in bees, similar to what is observed in vertebrates [64,65]. Together with SOD, these enzymes can detoxify reactive oxygen species through H_2_O_2_ intermediates to maintain redox homeostasis [59], and SOD activity has previously been shown to increase after fungicide exposure in honey bees [66].

The parallel dose–response experiments did not match the outcomes of the time-course experiment exactly, but the GST function was increased in response to increasing concentrations of Chunmanchun^®^. GST is one of the three classes of detoxifying enzymes [67,68] that we assessed and presumably is induced by either or both of the active Chunmanchun^®^ ingredients. However, we did not measure the corresponding mRNA transcript levels to confirm a potential induction of gene expression. POD activity was also significantly increased by the highest concentration, in contrast to CAT activity, which was significantly decreased. Dose- and time-dependent effects of fungicides on protective and detoxifying enzyme activities are common in honey bees [63,66], but our relatively low statistical power only allowed us to identify major effects. Other shortcomings of this assessment were our reliance on mass feeding, which presumably exposed different individuals to varying amounts of Chunmanchun^®^ and therefore increased within-group variation, and our reliance on newly emerged bees, which are still developing their physiological capacities and microbiomes.

We also studied the effects of different Chunmanchun^®^ doses on the honey bees’ gut microbiota because many bacterial species represent an initial line of defense against xenobiotics [69,70] and, in turn, can be disrupted by ingested pesticides [71,72]. Several differences were noted among the most common gut bacteria with increasing Chunmanchun^®^ exposure, although only *Lactobacillus* and *Bartonella* responded in a strict dose-dependent fashion. Other taxa responded more variably to Chunmanchun^®^, including *Snodgrassella*, which also belongs to the functionally significant core taxa [73,74]. While our study is the first to evaluate Chunmanchun^®^, the fungicide chlorothalonil also reduces the relative abundance of *Lactobacillus* with concomitant gains of most other taxa [75]. *Lactobacillus* can modulate honey bee learning and memory via regulating tryptophan metabolism [76], which may explain the reduced memory performance of Chunmanchun^®^ exposed bees. This explanation is corroborated by our finding of a dose-dependent imbalance in the Tyrosine metabolism pathway. Memory defects can decrease navigation and foraging performance with potentially cascading effects on colony health [77], although such measures were outside the scope of our study.

*Bartonella* was the second-most abundant taxon and exhibited the opposite trend, monotonously increasing with Chunmanchun^®^ exposure, although this effect was not statistically significant. Multiple species of *Bartonella* may coexist and the effect on honey bee health is unclear [78]. However, we cannot exclude the possibility that the fungicides directly benefit *Bartonella* or that this expansion is an indirect consequence of the reduction in *Lactobacillus* and other taxa. *Bartonella* seems to benefit from poor nutrition [79,80], which could suggest here that Chunmanchun^®^ lowers the nutritional value of otherwise similar diets taken in by the control and treatment bees. The low (PC) and the high (LD_50_), but not the intermediate (LD_10_) dose, of Chunmanchun^®^ increased *Snodgrassella* abundance significantly. This result may indicate important dose dependency. The interactive effects of another fungicide (azoxystrobin) with other stressors [81] make such non-linear effects plausible although their mechanistic basis remains unclear without further manipulative experimentation. Similarly to the other core bacteria, Snodgrassella is essential for honey bee health and metabolism [82,83] and it provides protection against opportunistic gut pathogens [84]. Even though we restrict our discussion to these three taxa, we cannot rule out that any of the additionally observed changes are functionally important. In addition, our metabarcoding data does not allow us to detect any potential changes within taxa or determine whether Chunmanchun^®^ affects the physiological functioning of the bees’ microbiome beyond compositional changes.

Based on the important metabolic roles of the gut microbiome [85], we predicted from the Chunmanchun^®^-induced changes in the microbiome that our four experimental groups would also differ in their gut metabolite composition. We found our prediction validated because metabolites from several pathways were consistently more abundant with increasing fungicide doses. Some of the most notable pathways were involved in energy metabolism, such as glycerophospholipid metabolism, the phospholipase D signaling pathway, and protein and fat digestion and absorption. Correspondingly, most of the differentially abundant metabolites were lipids. Such up-regulation of metabolism could be driven by gut microbiota [86] and help fuel the detoxification or repair of damage inflicted by toxins. One elevated lipid, lysophosphatidylcholine (18:1 (9Z)), may trigger an inflammatory response via the proinflammatory fatty acid arachidonic acid in vertebrates [87]. Accordingly, it has been identified as an etiological factor in chronic inflammatory diseases [88,89] and may trigger an equivalent response in honey bees.

Propiconazole affects cytochrome P450s [90], which are important in metabolizing xenobiotics, including pesticides [91], and our KEGG pathway analysis did indeed indicate that the drug metabolism–CYP450 pathway was inhibited by Chunmanchun^®^. This result is consistent with our enzyme activity assay, although those assays were performed with small sample sizes and the downward trend after Chunmanchun^®^ treatment was not statistically significant.

Our data revealed numerous correlations between gut microbial taxa and specific metabolites. Most striking were the significant, negative correlations of *Snodgrassella* with 70% of the key metabolites that were affected by Chunmanchun^®^. *Snodgrassella* was neither the most abundant nor the most consistently affected bacterial genus in our experiments, but its high correlation with a number of gut metabolites confirms nevertheless the importance of this taxon. In contrast, the abundance of *Gilliamella*, which forms a close association with *Snodgrassella* [74], was not correlated with any of the key metabolites in the gut, possibly indicating that *Snodgrassella*’s functions may be rate-limiting in this association. However, *Gilliamella* has associations with the carbohydrate and glycerophospholipid metabolites in the hemolymph of honey bees [92]. Other strong correlations were found for homovanillic acid, which was negatively associated with *Bartonella* sp. and positively with *Lactobacillus* sp. The microbiome strongly affects homovanillic acid with neurobiological consequences in honey bees [92] and other organisms, including humans [93], which could also explain our observed Chunmanchun^®^ effects on memory. However, with all of the identified correlations between metabolites and microbiota, we cannot establish any causation because specific metabolites may be the result of microbiome changes, but metabolites could also cause changes in the microbiome composition or both concomitant changes could be caused directly by Chunmanchun^®^ toxicity.

## 5. Conclusions

Our study found that Chunmanchun^®^ (35% carbendazim·propiconazole suspoemulsion) has low acute toxicity and poses little lethal risk to honey bees. However, we identified sublethal effects—including gut disturbances and cognitive impairments—that warrant further investigation. These findings indicate that Chunmanchun^®^ could negatively impact bee health beyond acute individual-level toxicity. We did not assess colony-level performance or field survival, which limits the practical interpretation of our results. Given the potential for broader ecological risks, particularly to solitary pollinators, we caution against the assumption that spraying Chunmanchun^®^ during flowering is without consequence. Our results underscore the need to go beyond standard toxicity assays, as sublethal effects may interact with other stressors under real-world conditions.

## Figures and Tables

**Table 1:** The details **of** all the chemical reagents used in our experiments.

Reagents	Manufacturer	Cat.# Number	Quantity
Chunmanchun^®^: 35% propiconazole·carbendazim suspoemulsion (7% propiconazole, 28% carbendazim) suspension–emulsion	Zhaoyuan Sanlian Chemical Factory Co., Ltd., Zhaoyuan, China	PD20111232	200 g
98.7% Dimethoate	Tanmo Quality Inspection Technology Co., Ltd., Shanghai, China	70529	100 mg
99.5% Acetone, analytically pure	Yonghua Chemical Technology (Jiangsu) Co., Ltd., Changshu, China	XK13-201-00115	500 mL
99.7% Absolute ethanol, analytically pure	Tianjin Yongda Chemical Reagent Co., Ltd., Tianjin, China	XK13-011-00011	500 mL
Citral	Shanghai Aladdin Biochemical Technology Co., Ltd., Shanghai, China	A2303116	25 mL
PBS solution	Bio-Channeln Biotechnology Co., Ltd., Shenzhen, China	BC20220606	500 mL
Superoxide dismutase (SOD) kit	Beijing Solarbio Science & Technology Co., Ltd., Beijing, China	BC0170	50T/24S
Peroxidase (POD) kit	Beijing Solarbio Science & Technology Co., Ltd., Beijing, China	BC0090	50T/48S
Catalase (CAT) kit	Beijing Solarbio Science & Technology Co., Ltd., Beijing, China	BC0200	50T/48S
Carboxylesterase (CarE) kit	Beijing Solarbio Science & Technology Co., Ltd., Beijing, China	BC0840	50T/48S
Glutathione-S transferase (GSTs) kit	Beijing Solarbio Science & Technology Co., Ltd., Beijing, China	BC0350	50T/48S
Insect cytochrome P450 (CYP450) enzyme-linked immunoassay kit	Shanghai Enzyme Biotechnology Co., Ltd., Shanghai, China	MI037418	48T/96T
Ultrapure water	Shanghai Kanglei Analytical Instrument Co., Ltd., Shanghai, China	1503106SN15VF	SMART-N
Sucrose	Jiangsu Baimei Sugar Industry Co., Ltd., Huaian, China	-	50 kg

## Data Availability

The gut microbiota data of honey bees have been deposited in the National Center for Biotechnology Information with the BioProject accession PRJNA1095985 (https://www.ncbi.nlm.nih.gov/bioproject/PRJNA1095985, accessed on 3 April 2024). The metabolomic data have been deposited in the National Genomics Data Center under the BioProject accession PRJCA024946, with the data identifier OMIX006155 (https://ngdc.cncb.ac.cn/omix/select-edit/OMIX006155, accessed on 5 April 2024). All other data supporting the findings of this study are available in the manuscript or Supplementary Materials.

## Supplementary Materials

The following supporting information can be downloaded at https://www.mdpi.com/article/10.3390/insects16060603/s1. Table S1: Acute oral toxicity in bees; Table S2: Effects of the compound fungicide on learning and memory ability of *Apis mellifera* (*n* = 20, 3 replicates for each group, extending the proboscis record as 1, not extending the proboscis record as 0); Table S3: Effects of the compound fungicide on memory of *Apis mellifera* analyzed by Chi-square Test (mean ± SD, *n* = 20, 3 replicates for each group); Table S4: Effects of compound fungicide at a field recommended concentration (PC) (C= 0.159 g/L on the activities of protective and detoxifying enzymes of bees at different time points (mean ± SD, *n* = 3)); Table S5: Effects of different concentrations of compound fungicide on the activities of protective and detoxifying enzymes of bees; Table S6: The microbial midgut composition of four different treatments at ASV level; Table S7: Comparison of alpha diversity indices among different concentration of Chunmanchun; Table S8: Analysis of differences in intestinal flora of bees at the genus level after stress with different concentrations of compound fungicides; Table S9: Metabolites identified in the four different treatments after data preprocessing; Table S10: Differential metabolites between the PC and CK groups; Table S11: Differential metabolites between the LD_10_ and CK groups; Table S12: Differential metabolites between the LD_50_ and CK groups; Table S13: Differential metabolites enriched KEGG pathways between the PC and CK groups; Table S14: Differential metabolites enriched KEGG pathways between the LD_10_ and CK groups; Table S15: Differential metabolites enriched KEGG pathways between the LD_50_ and CK groups.
